# Evaluation of Four Pulpotomy Techniques in Primary Molars: A Randomized Controlled Trial

**DOI:** 10.22037/iej.v13i1.18407

**Published:** 2018

**Authors:** Ghassem Ansari, Seyyedeh Pouya Morovati, Saeed Asgary

**Affiliations:** a *Department of Pediatric Dentistry, Dental School, Shahid Beheshti University of Medical Sciences, Tehran, Iran;*; b *Department of Pediatric Dentistry, Dental School, Kurdistan University of Medical science, Sanandaj, Iran;*; c * Iranian Center For Endodontic Research, Research Institute of Dental Sciences, Dental School, Shahid Beheshti University of Medical Sciences, Tehran, Iran*

**Keywords:** Calcium-Enriched Mixture, CEM Cement, Ferric Sulfate, Formocresol, Low Level Laser Therapy, Primary Molar, Pulpotomy

## Abstract

**Introduction:**

This trial was designed to evaluate the clinical and radiographic success rates of calcium-enriched mixture (CEM) cement with and without low level laser therapy (LLLT) and compare them to that of formocresol (FC) and ferric sulfate (FS) in primary molar pulpotomies.

**Methods and Materials:**

This randomized clinical trial was conducted on a total of 160 teeth selected from 40 patients aged 3-9 years. Patients with at least four primary molars needing pulpotomy, were included in order to have each tooth assigned randomly in one of the four following groups; FC, FS, CEM, and LLLT/CEM. Six- and twelve-month follow-up periods were conducted in order to enable a clinical and radiographic evaluation of the treated teeth. Collected data were analyzed using Cochran Q Tests.

**Results:**

The 12-month clinical success rate for each technique was: FC=100%, FS=95%, CEM=97.5% and LLLT/CEM=100% with no significant differences (*P*>0.05). Furthermore, 12-month radiographic success rate for each technique was: FC=100%, FS=92.5%, CEM=95% and LLLT/CEM=100% with no significant differences (*P*>0.05).

**Conclusion:**

Favorable outcomes of four treatment techniques in pulpotomy of primary molar teeth were comparable. CEM with/without LLLT may be considered as a safe and successful pulpotomy treatment modality compared to current conventional methods.

## Introduction

Nowadays, pulpotomy continues to be the most common treatment for asymptomatic decayed primary molars with pulp exposure. The main target of this procedure is to preserve the involved primary tooth to its normal exfoliation stage while inflamed coronal tissue is removed [1, 2]. This process involves the use of medicaments capable of being bactericidal and free of any side effects while promoting the healing process. An ideal medicament used for pulp chamber filling should not interfere with physiologic root resorption [3].

Several materials have been proposed and used by clinicians including formocresol (FC), ferric sulfate (FS), calcium hydroxide (CH), sodium hypochlorite (SH), mineral trioxide aggregate (MTA), and more recently calcium-enriched mixture (CEM) in pulpotomy of primary molars [4]. Among these, FC has long been used as the material of choice for pulp therapy in primary molars. However, due to the potential systemic spread of FC molecules through the root canals [5] causing toxicity, hypersensitivity and teratogenicity [6, 7], replacement with a safe medication is highly essential. In this regard FS has been tested and showed degrees of success through formation of a protein complex that occludes the capillary orifices in order to shape the blood clot and reduces the risks of inflammation and subsequent internal resorption [8]. A relatively high success rate has also been reported for the use of MTA in primary molar pulpotomy, while technique sensitivity, staining and high expenses make its use unfavorable in certain cases [9].

**Figure 1:**
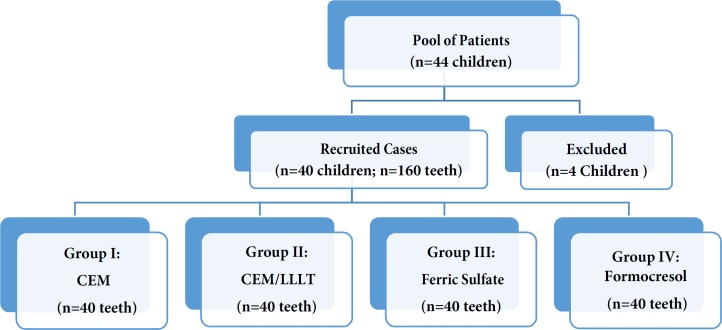
Flowchart detailing the number of patients recruited and those excluded from this investigation

CEM cement has been successfully tested in several studies on permanent teeth [10, 11]. The effectiveness of this biomaterial in primary molar pulpotomy has also been evaluated with results indicative of promising outcomes [12, 13]. Almost all medications used have some impact on the remaining pulp tissue in order to preserve the vitality of the pulp except FC which induces fixation of underlying tissue. The philosophy behind the use of FC was to disinfect and remove the remaining inflammatory cells from the area. 

As FS, MTA or CEM have similar potentials, the use of lasers for pulpotomy was advocated to overcome this issue when used along with one of the other materials named before. In this line low level laser therapy (LLLT) has been successfully tested as promoting the healing process in human cells. This is while the high power lasers have been used to remove caries as well as amputating the pulp while forming a clot layer at the cut surface. The use of various laser energies is on the rise for pulp application in pediatric dentistry [14, 15]. Various laser wave lengths have been demonstrated as being safe, effective and non-toxic alternatives for pulpotomy procedure of primary teeth [16, 17]. The use of LLLT has been mainly focused on tissue healing acceleration. Its effect on remaining pulp tissue is yet to be evaluated following amputation and homeostasis in primary teeth [18, 19]. 

This investigation aimed to compare the clinical and radiographic effectiveness of CEM cement with and without LLLT to FC and FS in pulpotomy of primary teeth.

## Materials and Methods

This randomized clinical trial was carried out on a group of healthy children aged 3-9 years. Forty-four cases were recruited among which 42 were included that met the inclusion criteria from a large pool of patients referred to the Dental Department of Mofid Children Hospital at Shahid Beheshti University of Medical Sciences, Tehran, Iran for dental treatments under general anesthesia (Figure 1). Selected children had at least four molar teeth needing pulpotomies in four quadrants with teeth allocated to one of the four groups in a random sampling manner. Selection criteria include: carious teeth with vital pulp exposure, no clinical or radiographic evidence of pulp degeneration, no excessive bleeding, no pathologic mobility, no swelling or fistula, no history of spontaneous and nocturnal pain, and tenderness to percussion or palpation, no external or internal root resorption, no inter radicular or periapical radiolucency. Only teeth having no more than one third of their roots undergoing physiologic resorption were included [20-22].

Fearful children who had no history of systemic disease, developmental problems or on any type of medication were included in this investigation. Patients were scheduled and seen under general anesthesia where all teeth received treatment at the same session and under almost unified circumstances. All procedures performed in this investigation were in accordance with the ethical standards of the institutional and national research committee and with the 1964 Helsinki declaration and its later amendments or comparable ethical standards. An informed consent form was given to the parents to read and sign prior to the operation.

Preoperative periapical radiographs were obtained from each tooth before treatment. Complete caries removal was performed using a large round carbide bur on a slow speed handpiece followed by access opening to the pulp chamber using a No.330 diamond bur (Tizkavan, Tehran, Iran) on a high-speed handpiece with water spray before coronal pulp tissue being removed by a sharp spoon excavator. Homeostasis was obtained through packing a sterile, saline-wet cotton pellet on the radicular pulp stumps with a gentle pressure. In case of problem with homeostasis, the tooth was excluded from the study and replaced. 

These steps were followed in all teeth with the rest of the steps differe in each group as: Group I received CEM (BioniqueDent, Tehran, Iran) with a 2 mm thickness covered with reinforced ZOE (Zonalin, Kemdent, UK), Group II received LLLT (Diode laser 632nm; Mustang 2000, Russia) application in continuous mode with total energy of 4.0 J/cm^2^ at 135 seconds exposure followed by CEM placemen and Zonalin on top, Group III received a moistened cotton pellet with FS (15.5% solution; Astringedent, Ultradent Products Inc., UT, USA) for 15 sec then removed and Zonalin was placed on top, and Group IV received a moistened cotton pellet with diluted FC (5:1 ratio; Sultan Chemists, Inc. Englewood, NJ, USA) for 5 min then removed followed by placement of Zonalin on top. Every tooth in each group was finally restored with stainless steel crown. 

Children were recalled for clinical and radiographic examinations at six and twelve months. Teeth that exhibited no symptoms of pain, tenderness to percussion, swelling, fistula or pathological mobility were judged clinically successful. Teeth that showed no evidence of periradicular or inter radicular radiolucency, internal or external root resorption, or periodontal ligament space widening were judged as radiographically successful. Clinical and radiographic outcome assessments were made by two independent calibrated pedodontists who were blind to the treatment groups. Statistical analysis was conducted using Cochran *Q* test on SPSS version 18.0 (SPSS Inc., Chicago, IL, USA) for groups comparison.

## Results

A total of 44 children were included among which 4 were excluded and calculations were carried out on remaining 40. These cases provided 160 first and second molars including 41 (25.6%) upper first molars, 55 (34.4%) lower first molars, 25 (15.6%) upper second molars and 39 (24.4%) lower second molars. The mean age of the patients was 4.6 (±0.6) years. 

Two cases in FS group had degrees of mobility and presented a fistula, one at 6-month and another case at 12-month follow-ups, both cases were judged as failed; while one case in CEM group had clinical signs of pain and mobility at 12 months (Table 1). However there was no significant difference between the clinical success rate of the four test groups at 6 (P=0.392) and 12 months (*P*=0.392). 

There was one case at 6 months and two at 12 months in FS group with radiographic signs of internal resorption associated with periapical radiolucency indicative of failure. There were also two cases in CEM group with degrees of external resorption and signs of furcation radiolucency at 12 months follow-up, both signs indicating failure (Table 2). However, statistical analysis did not show any significant difference between the radiographic outcome of all four groups at 6 (*P*=0.101) and 12 months of follow-up (*P*=0.392). 

Overall comparison between groups indicated no significant difference between the clinical and radiographic success rate of the four groups after 12-month follow-up using Cochran *Q* test (*P*>0.05).

## Discussion

Despite the high rate of reports on various types of medications and techniques for pulp treatment of primary teeth there are still gaps in various aspects of the procedure with no consensus. This includes the status of remaining pulp tissue after pulpotomy and indication of the technique appropriateness allowing the tissue to remain alive. Recent studies have focused on the alternative material to cover the remaining pulp while pulp amputation method has also been under investigation too. Among all studies performed there are those with strength in concluding statements based on the soundness and appropriateness of the methodology including proper case selection, randomization and fare judgment [3, 8]. This has a clear effect on the outcome reported which in turn influences its clinical implication. Antibacterial property, biocompatibility and non-toxicity are the main essential characteristics of medication material that comes into direct contact with the remaining pulp [23].

Despite all benefits of FC which was considered as the material of choice for many years, its potential hazards has raised concerns in recent years highlighting the need for a suitable successor [6, 7, 24]. Current study compared the effectiveness of CEM cement with and without the use of LLLT on the pulp remnants to that of FC and FS. Both first and second primary molars of the jaws were included in this trial in order to enable this comparison on various pulp configuration and supplies. In the same line Shirvani and Asgary reported no evidence for association between type of the cases selected (first/second molar, upper/lower jaw), gender, and age with the treatment success rate; all of the treated teeth are recommended to be restored with SSC as a more reliable restoration with higher longevity [25]. Despite minor differences, results indicated that no significant difference could be established between groups in both clinical and radiographic evaluation steps at 6 months of follow-up. This was further confirmed by the repeated evaluation of these cases after 12 months with no significant difference to indicate any superiority or inferiority of the techniques over each other. 

In regards to the use of FS as a potential alternative to FC in pulpotomy of primary teeth, it is believed that this medication has the potential to induce hemostasis with no harmful effect on the remaining vital tissue of the pulp remnants [3, 26]. Interestingly, results of the current investigation did not show any significant difference between FS and FC either. In fact there are enough evidence to suggest that currently available materials including MTA, FS, and CEM are clinically acceptable alternatives to the FC with the last as having a promoting effect on the pulp tissue healing and repair particularly when associated with the LLLT. Sonmez *et al.* [8] indicated no significant difference between the outcome of FS and FC primary molar pulpotomies. Researchers confirmed earlier reports with no difference between the success rate of alternate materials to FC in pulpotomy of primary teeth [26, 27]. Fuks *et al.* [20] stated no statistically significant difference between those treated with FC and FS in the clinical and radiographic evaluation. Fei *et al.* [28] reported a higher overall success rate for FS compared to that of FC over a period of 12 months. In this trial, the clinical and radiographic success rate for FS pulpotomy was recorded as 95% and 92.5% after 6- and 12 months, respectively. There was however, a higher number of radiographic failure rate in FS treated cases, a difference which can be interpreted by the fact that it’s mechanism of action is different. FC causes fixation of the underlying tissue while FS can only help in hemostasis which has a higher risk of inflammation in longer terms [29].

**Table 1:** Clinical assessment of the four treatment groups at six- and twelve-month follow-up visits

**Treatment**	**Clinical**	**6 months**	**12 months**
		**N (%)**	**N (%)**
**Formocresol**	Success	40 (100)	40 (100)
	Failure	0 (0)	0 (0)
**Ferric Sulfate**	Success	39 (97.5)	38 (95)
	Failure	1 (2.5)	2 (5)
**CEM Cement**	Success	40 (100)	39 (97.5)
	Failure	0 (0)	1 (2.5)
**CEM/LLLT**	Success	40 (100)	40 (100)
	Failure	0 (0)	0 (0)

With the development of more recently introduced highly biocompatible materials such as MTA and CEM, pulp therapy of primary carious teeth has been revolutionized as they remove the dangers and side effects associated with the use of FC in children. MTA has proved to be a highly acceptable pulp capping agent. Human studies showed less inflammation and necrosis on the underlying vital tissues. Formation of a dentinal bridge and more frequent odontoblastic layer makes it advantageous to CH [30, 31]. Comparable result of MTA pulpotomy had been reported with FC indicative of its potential applicability in children as a replacement [1]. However, more investigations are needed to confirm MTA as a successful replacement as recent studies challenge the level of evidence available to support its clinical use in primary molar pulpotomy [32]. Being technique sensitive along with the potential tooth discoloration and high expenses makes it less likely to become routinely used for primary molar pulpotomy [33, 34].

CEM cement had been tested with degrees of success in primary molar pulpectomy [35] and pulpotomy as well as treatment of (im)mature permanent teeth [36, 37]. Earlier studies have evidences indicating high bio-stimulation capacity in line with the reproduction of dental hard tissue when CEM is in close contact with live viable pulpal structure [38]. Malekafzali *et al.* [12] found no significant difference between clinical and radiographic outcomes of MTA and CEM in pulpotomized teeth after 24 months. Physical, chemical and biological compatibility of CEM cement makes it a suitable replacement medication for pulpotomy in primary molars [38].

The current study showed high clinical and radiographic success rate for those treated with CEM with no significant difference when compared to those received FC after a year. Comparing the effect of LLLT on the remaining pulp after pulpotomy and before placement of CEM with those without laser irradiation did not reveal any significant clinical or radiographic difference. Interestingly a four group comparison at the same one year stage revealed that despite small number of failures in two groups of FS and CEM, no statistically significant differences could be detected between groups confirming the safe use of the newly tested techniques. Histological investigations on pulp reaction to these techniques would be suggested as the next step in order to see if there is any difference in pulp reaction at the histological level.

**Table 2:** Radiographical assessment of the four treatment groups at six- and twelve-month follow-up visits

**Treatment**	**Radiography**	**6 months**	**12 months**
		**N (%)**	**N (%)**
**Formocresol**	Success	40 (100)	40 (100)
	Failure	0 (0)	0 (0)
**Ferric Sulfate**	Success	39 (97.5)	37 (92.5)
	Failure	1 (2.5)	3 (7.5)
**CEM Cement**	Success	40 (100)	38 (95)
	Failure	0 (0)	2 (5)
**CEM/LLLT**	Success	40 (100)	40 (100)
	Failure	0 (0)	0 (0)

In more recent years, application of lasers has gained a considerable attention in dentistry with its various types including LLLT. It generally is delivered with the mean power of equal or less than 500 mw aiming to improve the healing process at cell level. In the case of pulpotomy its use focuses on encouraging the amputated pulp tissue to heal quicker and eliminates inflammation risks. It is therefore considered as a complementary step to the pulpotomy process in primary teeth. In addition, laser irradiation can enhance formation of calcified nodules in human dental pulp cells, as well as increasing in alkaline phosphatase activity helping the production of collagen and osteocalcin [39]. The positive effect of LLLT on reactional dentinogenesis induction in human teeth has been shown earlier with GaAlAs laser energy density of 4 J/cm^2^ and wavelength of 670 nm causing bio-modulation in pulp cells [40]. Nagasawa *et al.* [41] showed that both Argon laser and Nd:YAG had strongly stimulated the formation of secondary dentin when low level radiation of these wavelengths were applied.

Researchers revealed no difference between Laser/MTA and FC treated cases after 15 months [22]. Similar results were reported when two techniques of FC and Diod laser pulpotomy were compared [18]. Vahid Golpaygani *et al.* [18] stated that LLLT can be used successfully as a complementary step to conventional pulpotomy procedure in order to help the healing process in radicular pulp tissues while no such effect is expected when FC is used.

Generally the laser energy of 2 to 4 J/cm^2^ LLLT is advised to be employed for intra-oral soft tissue applications while powers of 4 to 10 J/cm^2^ is mostly applied on hard dental tissues and certain cases of extra-oral applications [42]. Result of two recent meta-analysis are indicative of the fact that 632 nm wavelength has been associated with the highest positive treatment effects on tissue repair. It has also been concluded that pinpoint introduction of such wavelength is most beneficial to the tissue healing. [43].

## Conclusion

Comparing FS, FC, CEM and CEM/LLLT pulpotomy techniques in treatment of primary teeth did not show any significant difference in their clinical and radiographic success in 6 and 12-month follow-ups. The use of LLLT has the power to promote healing pulp stumps while the use of CEM will encourage this healing process. These indicate that successful potential use of the novel method can be safely considered.
